# The impact of perioperative red blood cell transfusions in patients undergoing liver resection: a systematic review protocol

**DOI:** 10.1186/s13643-016-0217-5

**Published:** 2016-02-29

**Authors:** Sean Bennett, Laura Baker, Risa Shorr, Guillaume Martel, Dean Fergusson

**Affiliations:** Division of General Surgery, Department of Surgery, University of Ottawa, Ottawa, ON Canada; School of Epidemiology, Public Health and Preventive Medicine, Faculty of Medicine, University of Ottawa, Ottawa, ON Canada; The Ottawa Hospital, Ottawa, ON Canada; Clinical Epidemiology Program, Ottawa Hospital Research Institute, Center for Practice Changing Research Building, The Ottawa Hospital—General Campus, 501 Smyth Road, PO Box 201B, Ottawa, Ontario Canada

**Keywords:** Liver resection, Hepatectomy, Blood transfusion, Outcomes, Survival

## Abstract

**Background:**

Liver resection is commonly performed for malignant and benign disease and is associated with frequent use of intraoperative and postoperative blood transfusions. Blood transfusions are potentially life-saving, but they have many adverse effects; some well understood, and others less so. Some of the poorly understood side effects include increased risk of postoperative complications and possibly worse oncologic outcomes. The objective of this systematic review is to provide estimates of transfusion prevalence and the effects of perioperative blood transfusion on postoperative mortality and morbidity and long-term cancer outcomes in patients undergoing liver resection.

**Methods/design:**

The Cochrane, Medline, and EMBASE databases will be searched for any randomized controlled trial or observational cohort study comparing liver resection patients that received intraoperative or postoperative allogeneic red blood cell transfusions to those who did not. Outcomes include postoperative mortality, postoperative morbidity (infectious, liver failure, renal failure, cardiovascular/cerebrovascular events, and thromboembolic events), and long-term disease-free and overall survival. Only studies with adult, human patients (>18 years old) undergoing liver resection, in which the primary intervention of interest is blood transfusion will be included. Data will be extracted by two reviewers in duplicate and synthesized into a narrative review. Risk of bias will be assessed. When clinically and methodologically appropriate, meta-analysis will be performed.

**Discussion:**

Our review will synthesize the literature pertaining to the potential beneficial and detrimental effects of red blood cell transfusion in patients undergoing liver resection. It will be an important step in the development of guidelines for the appropriate use of blood transfusions in patients undergoing liver resection.

**Systematic review registration:**

PROSPERO CRD42015026132

**Electronic supplementary material:**

The online version of this article (doi:10.1186/s13643-016-0217-5) contains supplementary material, which is available to authorized users.

## Background

Liver resection is a major intraabdominal surgery performed for a number of indications, but most commonly for removal of malignant neoplasms. The liver receives approximately 25 % of the cardiac output [1], and therefore resection is associated with moderate or significant blood loss, at times life-threatening, and not uncommonly results in the use of intraoperative or postoperative blood transfusions. The loss of blood from the intravascular space results in decreased oxygen-carrying capacity and decreased delivery of oxygen to the tissues; however, numerous physiologic adaptations occur to cope with these changes. The amount of tolerable blood loss is difficult to define precisely and is influenced by individual patient and disease-related factors. The estimation of surgical blood loss is also difficult, and many different methods and calculations of blood loss have been studied and described [2, 3]. Furthermore, the decision to replace blood volume by way of blood transfusion is one that requires multiple pieces of clinical and laboratory-based information. Typically, intraoperative blood volume replacement is done by the anesthesiologist, but communication with the surgical team is critical. In making this decision, multiple factors are considered including the patient’s hemodynamic status, cardiovascular disease status, hemoglobin concentration, hematocrit, the estimated blood loss, and the perceived rate of blood loss [2].

Blood transfusions have been shown to suppress the immune system in a number of ways, including impaired natural killer cell cytotoxicity [4] and lymphocyte activity [5]. The immunomodulatory effects of transfusions were first highlighted clinically by Opelz et al. when they showed improved survival of kidney transplant grafts with increasing number of pretransplant transfusions [6]. There has been a number of observational, retrospective studies showing association between blood transfusion and infectious complications [7–10], as well as early cancer recurrence [11–13]. A 2012 Cochrane review of 36 studies demonstrated an increased odds ratio of 1.42 (95 % CI, 1.20 to 1.67) for recurrence of colorectal cancer in patients receiving perioperative blood transfusions [12]. Despite this, there is much conflicting evidence in the literature pertaining to blood transfusions and cancer recurrence. This remains an area where blood transfusions have a perceived negative consequence without high-quality evidence to support such a claim.

Blood transfusions are administered to approximately one third of patients undergoing liver resections [14–18], and this rate appears to be decreasing over time. In a series of 1351 patients undergoing liver resection for colorectal liver metastases between 1986 and 2001 at a single center, Kooby et al. found that 55 % of patients received a blood product transfusion (red blood cells, platelets, or plasma) either intraoperatively or during their postoperative hospitalization [10]. They also demonstrated a reduction in the use of blood products over time, with 83 % of patients between 1986 and 1990, 54 % of patients between 1991 and 1994, and 43 % of patients between 1995 and 2001 receiving blood. They found that non-transfused patients experienced fewer serious postoperative complications (33 vs 46 %) and that patients transfused more than 2 units of blood experienced more complications than those transfused 1 or 2 units (51 vs 42 %). Blood transfusion remained a significant predictor of complication after multivariate analysis (OR 1.5), along with larger resections and male gender. Blood transfusion was also found to be an independent predictor of 60-day mortality on multivariate analysis (OR 3.7), but not to have a significant impact on long-term survival beyond the 60-day postoperative period. More recent data demonstrates that the trend towards less blood transfusion continues. In a study of 2448 patients undergoing liver resection in 2013, the rate of blood transfusion for the entire cohort was 22.1 % [19].

## Objective

The primary objective of this review is to synthesize the evidence surrounding the prevalence and impact of intraoperative and postoperative transfusions of allogeneic red blood cells on key clinical outcomes in patients undergoing liver resection. The key clinical outcomes of interest include transfusion prevalence, postoperative mortality, postoperative morbidity (infectious complications, liver failure, acute renal failure, cardiovascular and cerebrovascular events, and thromboembolic events), and long-term overall and disease-free survival.

## Methods/design

Our systematic review was designed using the Preferred Reporting Items for Systematic Review and Meta-Analyses (PRISMA) guidelines [20]. A PRISMA-Protocol checklist was followed (see Additional file 1). The protocol has been registered with the PROSPERO International Prospective Register of Systematic Reviews (CRD42015026132).

### Eligibility criteria

#### Population

The population of interest is adult patients, over the age of 18, undergoing elective liver resection for any indication. This does not include patients having emergency liver resection for trauma or bleeding. It does include patients undergoing partial liver resection in order to be a transplant donor but does not include patients receiving liver transplants. Studies that include liver resection in addition to other procedures will be included if the liver resection data can be extracted from the rest, either from the paper directly or through communication with the corresponding author.

### Intervention

The intervention being studied is the administration of allogeneic red blood cell transfusions during the liver resection (intraoperative), or during the immediate hospitalization following liver resection (postoperative). This review will not focus on the administration of other types of blood products, such as autologous transfusions, platelets, plasma, or cryoprecipitate.

### Comparators

Any randomized controlled trial (RCT) or observational cohort study comparing patients who received red blood cell transfusions to those who did not will be included in this review.

### Outcomes

The outcomes of interest include transfusion prevalence, postoperative mortality, postoperative morbidity, and long-term cancer survival outcomes. As postoperative mortality can be defined at a number of time points, all will be acceptable and will be categorized and described in the review. Postoperative morbidities that will be included are overall morbidity, infectious complications (surgical site infection, pneumonia, urinary tract infection), acute liver failure/insufficiency, acute renal failure, cardiovascular events, cerebrovascular events, and thromboembolic events. Severity of complications will be considered and categorized in subgroups, using the Clavien-Dindo classification, if available from the reported data. Long-term cancer survival outcomes will include overall survival and disease-free survival, as well as disease recurrence. Studies that report any, or all, of these outcomes will be considered for inclusion.

### Search strategy

The search strategy was created by the primary investigator (SB) and an expert medical librarian (RS). The Medline (1946–present), Embase and Embase Classic (1947–present), and the Cochrane Central Register of Controlled Trials will be searched from inception until September 2015 for articles using a combination of MESH and text words for liver resection and blood transfusion (see Additional file 2). Searches are restricted to human studies with adult (>18 years old) patients. Study inclusion will be limited to titles written in English or French. Reference lists will be reviewed for additional studies. Gray literature and conference proceedings will not be searched specifically, although abstracts identified in the search strategy will be considered for inclusion to minimize the impact of publication bias. To avoid duplicate study selection, author names will be compared, and if there is uncertainty, corresponding authors will be contacted.

### Study selection

After a pilot screening evaluation conducted by two independent investigators (SB and LB) of 100 titles and abstracts to establish excellent agreement (kappa > 0.75), one investigator (SB) will conduct the initial title and abstract screen for the remainder. Two investigators (SB and LB) will then conduct the full text review to identify studies meeting inclusion/exclusion criteria. Disagreements will be resolved via consensus where possible, and by a third reviewer, if necessary (GM).

### Data extraction

Two investigators (SB and LB) will extract data in duplicate from the included studies into a spreadsheet developed a priori. Data will include publication details, study design, study size, patient demographics, outcomes used, confounding variables controlled for on multivariate analysis, and effect of treatment on outcomes.

### Quality assessment

Two investigators (SB and LB) will independently assess the included studies for risk of bias and quality of reporting. Disagreement will be resolved via consensus or a third investigator, when necessary. The included studies are expected to all be observational cohort studies, with no RCTs. Should there be any RCTs, they will be assessed using the Cochrane Handbook “Risk of Bias” assessment tool [21]. The observational cohort studies will be assessed using the Cochrane Risk of Bias Assessment Tool for Non-Randomized Studies of Interventions (ACROBAT-NRSI) [22]. This will evaluate risk of bias due to confounding, selection, measurement, and interpretation. The quality of reporting will be assessed using the strengthening the reporting of observational studies in epidemiology (STROBE) checklist [23].

### Data synthesis

The data will be entered and interpreted into a narrative synthesis. The narrative synthesis will be grouped by outcome of interest (postoperative mortality, postoperative morbidity, and long-term cancer outcomes). Within each outcome group, the studies will be subgrouped by liver pathology (primary liver tumors, metastatic disease, and donor hepatectomy), as these each have their own distinct clinical characteristics when it comes to blood transfusion. Other subgroupings of interest may include date of publication, methodological quality, and extent of adjustment for confounding.

It is unlikely that a quantitative meta-analysis will be feasible due to expected lack of controlled clinical trials and clinical and statistical heterogeneity; however, this will be considered once the data is collected. Where possible, given the availability of data, and the clinical and statistical homogeneity, a quantitative meta-analysis of observational studies may be performed. If a meta-analysis is possible, the effect of blood transfusion will be stratified by outcome, using a random effects model. We will also evaluate the impact of important clinical and methodological characteristics through the conduct of subgroup analyses. Planned subgroup analyses include indication for surgery (benign, primary malignant, metastatic), risk of bias score, and method of multivariate analysis. We will also explore sources of potential heterogeneity through visual inspection of overall pooled analyses and inspection of *Q* and *I*^2^ statistics. Quantitative meta-analysis will be performed using OpenMetaAnalyst software (Brown University, School of Public Health).

## Discussion

There have been a number of recent calls for guidelines on the appropriate use of blood transfusions during liver resection [1, 24]. Given the potential life-saving benefit of blood transfusions, as well as the possibility of negative clinical consequences when given inappropriately, this is an important future direction. While general guidelines on perioperative blood management do exist [25], none are specific to liver surgery. Furthermore, there is published evidence of practice variability in the use of blood conservation methods during liver resection [26], as well as in the use of blood transfusions during liver transplant [27].

A thorough synthesis of the current body of literature is a necessary initial step in this process. One previous systematic review and meta-analysis showed blood transfusions had increased postoperative mortality, postoperative morbidity, and cancer recurrence after liver resection but only studied patients with hepatocellular carcinoma [28]. The proposed review will expand the patient population to include patients undergoing liver resection for all indications, in particular for colorectal liver metastases, which is the most common indication for liver resection in North America [19, 29]. Furthermore, this previous review included studies up until 2012, and an update to include the past three years of literature will be important.

The strengths of the proposed systematic review will be a very broad search strategy, rigorous inclusion/exclusion criteria, a focus on only studies where blood transfusion is the primary intervention of interest, and the inclusion of all indications for elective liver resection. Limitations of this review will likely include a lack of RCTs and significant clinical and statistical heterogeneity between studies. This will likely prevent the conducting of a quantitative meta-analysis.

## Additional files

Additional file 1: PRISMA-Protocol Checklist. (DOC 82 kb) Additional file 2: Example of the Medline search strategy. (PDF 25 kb)

## Abbreviations

FFP: fresh frozen plasma
pRBC: packed red blood cells
PRISMA: Preferred Reporting Items for Systematic Review and Meta-Analyses
RCT: randomized controlled trial
STROBE: strengthening the reporting of observational studies in epidemiology
