# Supplementary material for: The impact of perioperative red blood cell transfusions in patients undergoing liver resection: a systematic review protocol
Source: Syst Rev. 2016 Feb 29;5:38. doi: 10.1186/s13643-016-0217-5 (PMC4770706; doi:10.1186/s13643-016-0217-5)
Supplement: Additional file 2: — Example of the Medline search strategy. (PDF 25 kb) [file 13643_2016_217_MOESM2_ESM.pdf]

Database: Ovid MEDLINE(R) In-Process & Other Non-Indexed Citations and Ovid MEDLINE(R) <1946 to Present>
Search Strategy:
----
1 exp Liver Neoplasms/su (20551)
2 Hepatectomy/ (23248)
3 hepatectom*.tw. (17035)
4 ((liver or hepatic or hepato*) adj2 resect*).tw. (14735)
5 or/1-4 (44945)
6 exp blood transfusion/ (85009)
7 ((rbc or blood$ or red cell$ or erythrocyte$ or plasma or platelet$) adj2 transfusion$).tw. (46072)
8 transfusion*.ti. (32101)
9 cryoprecipitate.tw. (1736)
10 or/6-9 (111774)
11 5 and 10 (1280)
12 remove duplicates from 11 (1264)
